# Circ_0044516 Regulates miR-136/MAT2A Pathway to Facilitate Lung Cancer Development

**DOI:** 10.1155/2021/5510869

**Published:** 2021-06-24

**Authors:** Yue-Wei Chen, Qiu-Rong Du, Yu-Juan He, Wen-Shu Chen, Wen-Yang Jiang, Qi Gui, Cheng-Cheng Xu, Wei Wang, Hong-Yun Cheng

**Affiliations:** ^1^Department of Cardiothoracic Surgery, Hospital of Chengdu University of Traditional Chinese Medicine, Chengdu, China; ^2^Department of Clinical Laboratory, Hospital of Chengdu University of Traditional Chinese Medicine, Chengdu, China; ^3^Department of Thoracic Surgery, Shengli Clinical Medical College of Fujian Medical University, Fuzhou, China; ^4^Department of Thoracic Surgery, Renmin Hospital of Wuhan University, Wuhan, China; ^5^Department of Thoracic Surgery, The First Affiliated Hospital of Soochow University, Suzhou, China; ^6^Department of Oncology, The First Affiliated Hospital of Soochow University, Suzhou, China; ^7^Department of Oncology, Huai'an Second People's Hospital and the Affiliated Huai'an Hospital of Xuzhou Medical University, Huai'an, China

## Abstract

Circular RNA (circRNA) is a type of noncoding RNA that can interact with miRNAs to regulate gene expression. However, little is known concerning circRNA, which is crucial in the pathogenesis of lung cancer. To date, limited studies have explored the role of circ_0044516 in lung cancer progression. Recently, we observed that circ_0044516 expression levels were obviously elevated in lung cancer tissues and cells. A549 and SPCA1 cells were transfected with circ_0044516 siRNA. We observed that knockdown of circ_0044516 dramatically repressed cell proliferation, increased cell apoptosis, and repressed the cell cycle. Moreover, A549 and SPCA1 cell migration and invasion abilities were greatly repressed by circ_0044516 siRNA. Due to accumulating evidence demonstrating the vital role of cancer stem cells, their mechanism of involvement has drawn increasing attention in tumor progression and metastasis research. We also found that cancer stem cell properties were restrained by silencing circ_0044516 in A549 and SPC-A1 cells. Moreover, *in vivo* xenograft experiments showed that circ_0044516 downregulation reduced tumor growth. Mechanistically, in lung cancer and using bioinformatics, we demonstrated that circ_0044516 sponges miR-136 targeting *MAT2A*. Furthermore, rescue assays were carried out to identify that circ_0044516 modulates cell proliferation, invasion, and stemness by regulating miR-136 and *MAT2A* in lung cancer. In summary, our study revealed that the circ_0044516/miR-136/*MAT2A* axis is involved in lung cancer progression. Our findings may provide novel targets for diagnosis and therapeutic intervention in lung cancer patients.

## 1. Introduction

Lung cancer is a prevalent malignancy and is becoming an important factor in tumor-related deaths worldwide [1]. Nonsmall cell lung cancer (NSCLC) and small-cell lung cancer (SCLC) are two common subtypes of lung cancer [2]. Significant progress has been achieved in lung cancer therapy. However, the five-year overall survival rate is low [3]. In addition, the outcome of lung cancer patients with metastasis or recurrence is very poor [4]. Therefore, understanding the pathogenesis of lung cancer progression will be of great significance in the management of the disease.

Back-splicing with no 5′-3′ polarity or a polyadenylated tail also contributes to the generation of circular RNAs (circRNAs) [5–7]. circRNAs are significant posttranscriptional regulators of gene control. CircRNAs can function via the competing endogenous RNA (ceRNA) network by competitively sponging microRNAs to modulate mRNAs [8]. Recently, circRNAs have been shown to contribute to tumor development and are typically present in various cancers [9–11]. For example, circ_100395 can regulate miR-1228 and TCF21 signaling to repress lung cancer development [12]. CircNOL10 suppresses lung cancer by inducing transcriptional modulation of the human polypeptide family mediated by SCLM1 [13]. In addition, circ_100146 can serve as a tumor inducer by regulating miR-361-3p [14]. CircFGFR1 promotes lung cancer progression by sponging miR-381-3p [15]. Hence, investigating the effects of circRNAs in cancer may be beneficial for lung cancer. To date, circ_0044516 has not been investigated in lung cancer progression.

Increasingly, circRNAs are involved in lung cancer, and in our research, we explored the effects of circ_0044516. Furthermore, we reported that circ_0044516 contributes to lung cancer by modulating miR-136 and *MAT2A*, which indicated that the circ_0044516/miR-136/*MAT2A* axis could be a crucial therapeutic target in lung cancer.

## 2. Materials and Methods

### 2.1. Lung Cancer Tissue

Lung cancer tissue samples and normal tissues were collected from lung cancer patients at The First Affiliated Hospital of Soochow University from 2012 to 2018. Adjacent normal tissues were >5 cm from tumor tissues. All patients were diagnosed with primary lung cancer and received no preoperative radiotherapy, chemotherapy, targeted therapy, or immunotherapy. General clinical information and detailed pathological records were collected. All participants involved in this study provided informed consent before the study. This study was approved by the Medical Ethics Committee of the First Affiliated Hospital of Soochow University.

### 2.2. Cell Lines

Human lung bronchial epithelial BEAS-2B cells and five lung cancer cell lines (A549, SPCA1, H1299, H460, and H23) were obtained from the Type Culture Collection of the Chinese Academy of Sciences (Shanghai, China). Cells were maintained in RPMI-1640 medium with 10% FBS and 1% penicillin/streptomycin (complete medium, Gibco, Aukland, New Zealand) with 5% CO_2_ and 95% air at 37°C.

### 2.3. Cell Transfection

siRNAs of circ_0044516(siRNA-01-TTCCAGGGTCCCGCCGGTCAA; siRNA-02- GGATTCCAGGGTCCCGCCGGT), miR-136 mimics, circ_0044516, MAT2A overexpression plasmid, and negative controls (NCs) were purchased from GenePharma (Shanghai, China). Transfections (50 nM miRNA mimics, inhibitors, and siRNAs) were carried out using the Lipofectamine 3000 reagent (Carlsbad, Invitrogen, CA, USA) based on the protocols provided by the manufacturer.

### 2.4. CCK-8 Assays

Lung cancer cells were grown in 96-well plates and after transfection for 48 h, and CCK-8 solution (Beyotime, Shanghai, China) was added to the cells to measure cell proliferation. A microplate reader was used to test the optical absorbance values at 450 nm to assess cell proliferation.

### 2.5. Cell Apoptosis

Cells (2 × 10^5^) were collected in 1.5 mL EP tubes. The supernatant was discarded after centrifugation at 2,000 × *g* at 4°C. 500 *μ*L binding buffer was used to resuspend the cells. Annexin V-FITC (5 *μ*L) was added and incubated at 4°C for 30 min in the dark. Next, 5 *μ*L propidium iodide (PI) was gently mixed and incubated at room temperature for 5 min. The cell apoptosis rate was detected using an Annexin-V-FITC detection kit (K201-100, BioVision, USA).

### 2.6. Cell Cycle

In brief, cells were fixed with 70% alcohol overnight at -20°C. Afterwards, staining buffer was added to resuspend the cells. Then, PI staining solution was added to the resuspended cells for 1 min at 37°C with no light. Subsequently, a flow cytometer was used to analyze the samples.

### 2.7. Spheroid Formation

Cells under different treatments were seeded on ultralow attachment 24-well plates (Corning, Union City, CA, USA) at 1,000 cells/mL in 500 *μ*L volume and maintained in serum-free RPMI-1640 medium with 20 ng/mL EGF, B27, and 4 mg/mL insulin. Cells were cultured for 14 days and the medium was refreshed every 3 days followed by detection of the number and size of mammospheres.

### 2.8. Wound Healing Assays

Wound healing assays were performed to evaluate the migratory capacity of the cells. The wound healing capacity of lung cancer cells was tested. Cells were cultured in 6-well plates at up to ~100% confluence. Then, a 10 *μ*L pipette was employed to scratch a wound across the middle part of the well. Images of the wound were captured after 48 h.

### 2.9. Transwell Invasion Assays

Transwell chambers with Matrigel matrix were used to determine the invasive capacity of lung cancer cells. Briefly, cells (1 × 10^5^/ml) were collected and resuspended in serum-free culture medium. Next, the upper chamber was loaded with 200 *μ*L of the cell suspension. The lower chamber was filled with 500 *μ*L of culture medium. After 24 h, the invading cells were stained with 0.5% crystal violet.

### 2.10. Bioinformatics Analysis

Potential target miRNAs of circ_0044516 were predicted using the bioinformatics database tool CircNet and then further predicted by Shanghai Kangcheng Biotech, China. Potential target genes of miR-136 were predicted using TargetScanHuman (http://www.targetscan.org).

### 2.11. Western Blotting

The different groups of cells were lysed in lysis buffer (Beyotime, Shanghai, China) supplemented with protease inhibitors (Beyotime, Shanghai, China) and centrifuged. Protein concentrations were determined using a BCA kit (Pierce, Rockford, USA). After the separation by 10% SDS-PAGE, the proteins were transferred onto nitrocellulose membranes (Millipore, Billerica, USA). Nonfat milk (5%) was used to block the membranes. The membrane was then incubated with primary antibodies overnight. The primary antibodies included anti-MAT2A (1 : 5000, ab186129, Abcam) and anti-GAPDH (1 : 5000, ab3674, Abcam) antibodies. The next day, secondary antibodies were used. Protein bands were visualized using an enhanced ECL kit (Millipore).

### 2.12. qPCR

Total RNA was extracted using TRIzol reagent (Invitrogen). A NanoDrop 2000c (Thermo Scientific, Waltham, USA) instrument was used to assess RNA quality. A Bestar RT-qPCR Kit (DBI Bioscience, China) was used to generate cDNA. Bestar qPCR MasterMix was used to perform RT-qPCR on an ABI 7300 system. The primer sequences are shown in Table 1. The relative levels of gene expression were represented as ΔCt = Ct gene–Ct reference, and the -fold change in gene expression was calculated using the 2^−*ΔΔ*Ct^ method.

### 2.13. Dual Luciferase Reporter Gene Assays

Cells were seeded into 6-well plates and transfected as follows: pMIR-Reporter luciferase reporter plasmid containing wild-type (Luc-circ_0044516/MAT2A-WT) or mutated circ_0044516 3′UTR (Luc-circ_0044516/MAT2A-MUT) via Lipofectamine 3000 (Invitrogen), and transfected with miR-136 mimics. Luc-c/MAT2A-MUT with a mutated miR-136 binding site was constructed using the Site-directed Gene Mutagenesis Kit (Vazyme Biotech). After 72 h, luciferase activity was measured using a dual-luciferase reporter gene assay (Promega, Madison, WI, USA).

### 2.14. RNA Pull-Down Assays

To pull down circ_0044516, Biotin-labeled wild-type miR-136 or NC was used to pull down circ_0044516. Approximately 1 × 10^7^ lung cancer cells were lysed and incubated with a biotin-labeled miR-136 probe. The Pierce RNA 3′End Desthiobiotinylation Kit was used to label RNAs using biotin. Biotin-labeled wild-type miR-136 or NC was treated with cell lysates using magnetic beads. Streptavidin-coated magnetic beads were washed with lysis buffer, and Trizol (Takara) was used to purify the RNA complexes. The abundance of circ_0044516 was detected using RT-qPCR.

### 2.15. *In Vivo* Tumor Growth Assays

Xenograft assays were performed to analyze the role of circ_0044516 *in vivo*. Animal procedures were performed according to the Guide for the Care and Use of Laboratory Animals of the National Institutes of Health. Animal assays were approved by the Animal Care and Use Committee of the First Affiliated Hospital of Soochow University. 5-week-old female BALB/c nude mice were used for xenograft experiments and maintained under specific pathogen-free conditions. 5 × 10^6^ A549 cells transfected with circ_0044516 siRNA or NC were injected into 5-week-old female BALB/c nude mice. Tumor volumes were calculated using the formula (length × width2/2) and recorded weekly. After 28 days, the mice were sacrificed and tumor weights were determined. Tumor tissues were collected for further studies.

### 2.16. Statistical Analysis

SPSS v.19.0 software was used to carry out statistical analysis. All results are expressed as means ± SD. For comparison of two groups, two-tailed Student's *t*-test was performed. Multiple groups were compared using one- or two-way ANOVA. Statistical significance was set at *P* < 0.05.

## 3. Results

### 3.1. Elevated circ_0044516 Expression in Lung Cancer

**Figure 1:**
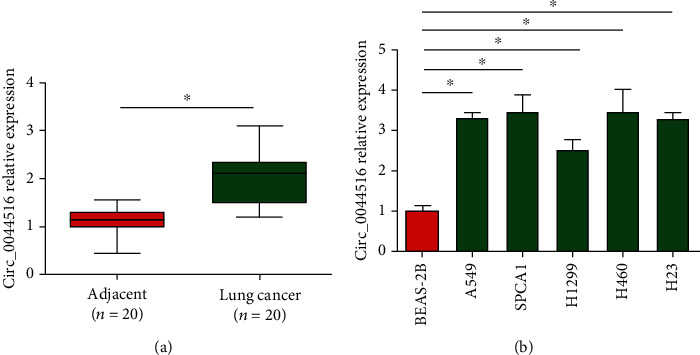
Upregulated circ_0044516 in lung cancer. (a) Circ_0044516 expression in 20 pairs of lung cancer tissues using real-time PCR. *GAPDH* was used as an internal control. (b) Circ_0044516 expression in A549, SPCA1, H1299, H460, and H23 cells and BEAS-2B cells. The data are presented as means ± SD. ^∗^*P* < 0.05.

First, to identify circ_0044516 expression in lung cancer, a total of 20 paired clinical lung cancer tissues and adjacent normal tissues were examined for circ_0044516 expression using RT-qPCR. We observed that circ_0044516 was upregulated in lung cancer tissues (Figure 1(a)). Next, we showed that circ_0044516 was elevated in lung cancer cells in comparison to Beas-2B cells, as shown in Figure 1(b). These results indicated that cir_0044516 expression was increased in lung cancer.

### 3.2. Effects of circ_0044516 siRNA on Lung Cancer Cell Proliferation, Apoptosis, and Cell-Cycle

**Figure 2:**
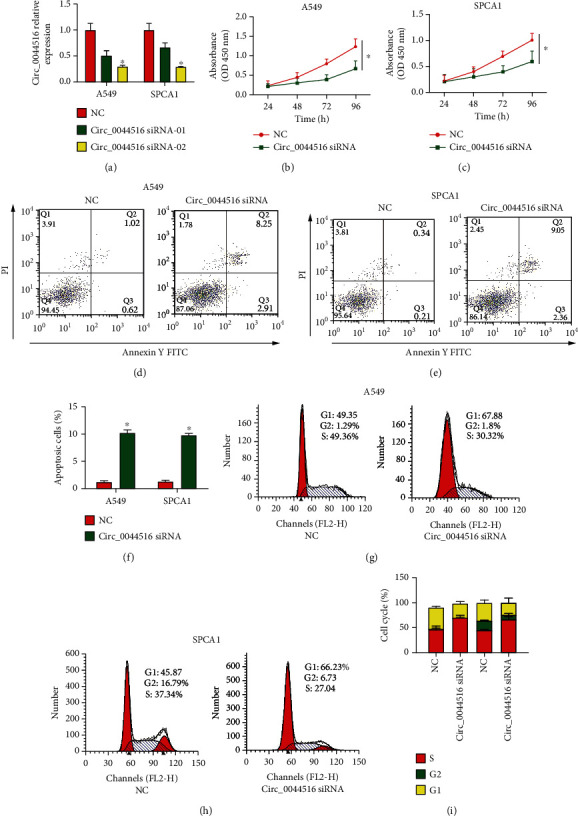
Effects of circ_0044516 siRNA on lung cancer cell proliferation, apoptosis, and cell-cycle. (a) Relative expression of circ_0044516 in A549 and SPCA1 cells transfected with circ_0044516 siRNA or siRNA control. (b, c) Downregulation of circ_0044516 reduced A549 and SPCA1 cell proliferation. CCK8 assays were carried out to assess cell viability. (d–f) Cell apoptosis was tested in A549 and SPCA1 cells using flow cytometric analysis. (g–i) Cell cycle distribution in A549 and SPCA1 cells using flow cytometry. Data are presented as means ± SD. ^∗^*P* < 0.05.

Circ_0044516 expression was highest in A549 and SPCA1 cells among the five lung cancer cell lines in our study. These two types of cells were transfected with two circ_0044516 siRNAs. In addition, circ_0044516 siRNA-02 exhibited a superior knockdown function; therefore, it was used for subsequent experiments (Figure 2(a)). Through CCK8 assays, loss of circ_0044516 repressed A549 and SPCA1 cell viability (Figures 2(b) and 2(c)). We then evaluated the effect of circ_0044516c on cell apoptosis and the cell cycle. Hochest analysis suggested that circ_0044516 downregulation resulted in increased cell apoptosis, as shown in Figures 2(d)–2(f). In addition, cell cycle distribution was blocked in the G0/G1 phase due to the lack of circ_0044516 (Figures 2(g)–2(i)). These data demonstrated that knockdown of circ_0044516 repressed lung cancer cell proliferation, enhanced cell apoptosis, and blocked cell cycle progression.

### 3.3. Effects of circ_0044516 siRNA on Lung Cancer Cell Migration and Invasion

**Figure 3:**
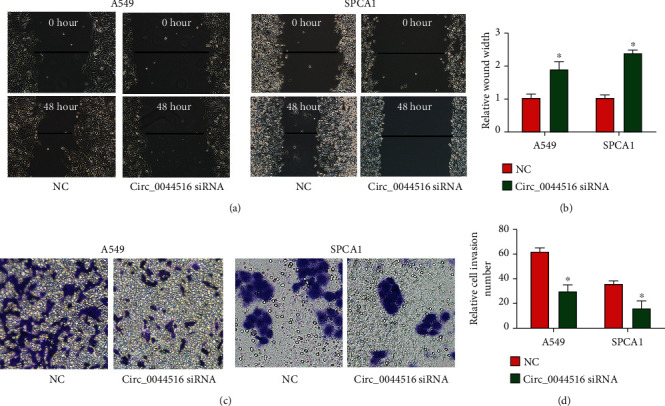
Effects of circ_0044516 siRNA on lung cancer cell migration and invasion. (a, b) Cell migration in A549 and SPCA1 cells transfected with circ_0044516 siRNA or NC. Wound healing assays were performed (c, d). Cell invasion was determined using transwell invasion assays. Data are presented as means ± SD. ^∗^*P* < 0.05.

A positive association between circ_0044516 expression and metastasis was observed. To confirm this, we performed wound healing and transwell invasion assays using A549 and SPCA1 cells. Circ_0044516 siRNA suppressed cell migration and invasion, as demonstrated in Figures 3(a)–3(d). These results indicated that silencing circ_0044516 might depress lung cancer cell migration and invasion.

### 3.4. Effects of circ_0044516 siRNA on Lung Cancer Stem Cell Properties

**Figure 4:**
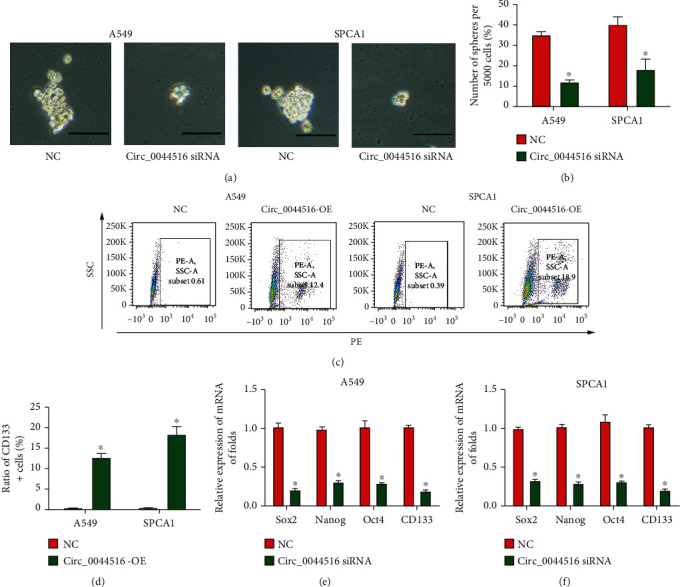
Effects of circ_0044516 siRNA on lung cancer stem cell properties. (a, b) Spheroid formation assays were carried out using A549 and SPCA1 cells transfected with circ_0044516 siRNA or NC. (c, d) Flow cytometric assays were used to evaluate the CD133-positive cell ratio in A549 and SPCA1 cells. (e, f) *Sox2*, *Nanog*, *Oct4*, and *CD133* mRNA expression. Data are presented as means ± SD. ^∗^*P* < 0.05.

To study the function of circ_0044516 on lung cancer stem cell properties, spheroid formation assays were carried out and circ_0044516 siRNA decreased sphere numbers in A549 and SPCA1 cells (Figures 4(a) and 4(b)). Moreover, flow cytometric assays indicated that the CD133 positive cell ratio was significantly elevated by increased levels of circ_0044516 in A549 and SPCA1 cells (Figures 4(c) and 4(d)). In addition, *Sox*, *Nanog*, *Oct4*, and *CD133* mRNA expression were reduced by circ_0044516 siRNA *in vitro* (Figures 4(e) and 4(f)). These findings implied that the loss of circ_0044516 restrained lung cancer stem cell properties.

### 3.5. Circ_0044516 Sponged miR-136

**Figure 5:**
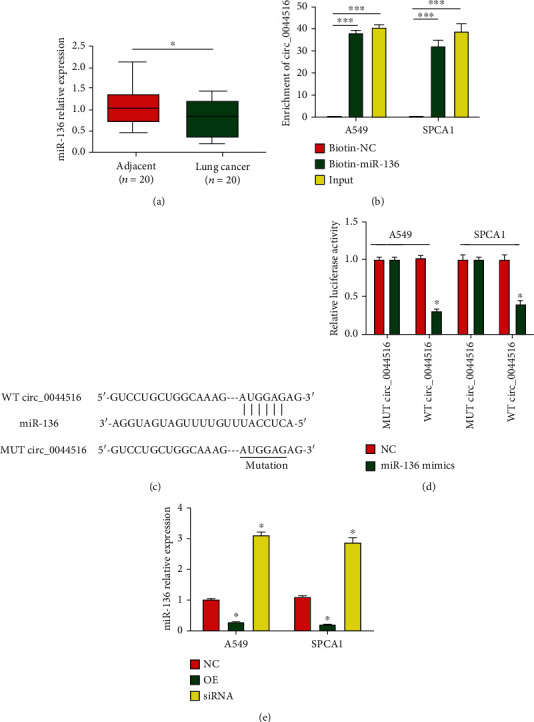
miR-136 is a direct target of circ_0044516. (a) miR-136 expression in 20 pairs of lung cancer tissues. (b) RNA pull-down assays were carried out using biotin-miR-136, control, or 10% input. (c) Predicted WT or MUT miR-136 binding sites in circ_0044516. (d) Relative luciferase activities were measured in A549 and SPCA1 cells cotransfected with circ_0044516-WT or circ_0044516-MUT and miR-136 mimics or miR-NC by luciferase reporter gene assays. (e) Expression of miR-136 in lung cancer cells transfected with circ_0044516 siRNA or circ_0044516 overexpression plasmid by RT-qPCR. Data are presented as means ± SD. ^∗^*P* < 0.05; ^∗∗∗^*P* < 0.001.

The miRNA targets of circ_0044516 were analyzed using https://circinteractome.nia.nih.gov/. miR-136 was predicted to be a target of circ_0044516. miR-136 levels were downregulated in lung cancer cells (Figure 5(a)). RNA pull-down assays using biotin-miR-136 were carried out using A549 or SPCA1 cell lysates. We found that circ_0044516 was enriched by biotin-miR-136 (Figure 5(b)). Next, WT-circ_0044516 and MUT-circ_0044516 were constructed (Figure 5(c)). The findings indicated that transfection with miR-136 mimics repressed the activity of WT-circ_0044516 *in vitro* (Figure 5(d)). In addition, miR-136 was negatively modulated by circ_0044516 in lung cancer cells (Figure 5(e)). Together, these data suggested that circ_0044516 could directly act as a sponge for miR-136.

### 3.6. *MAT2A* Is a Direct Target of miR-136

**Figure 6:**
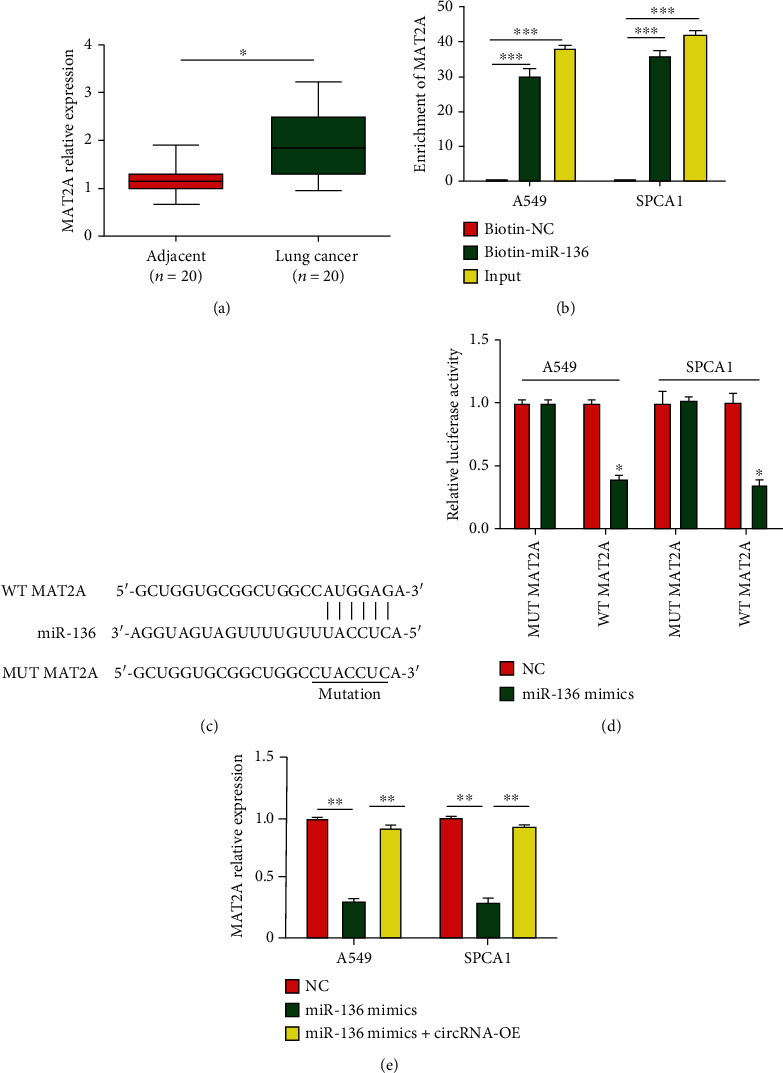
*MAT2A* is a direct target of miR-136. (a) *MAT2A* mRNA expression in 20 pairs of lung cancer tissues. (b) RNA pull-down assays were carried out with biotin-miR-136, control, or 10% input using A549 or SPCA1 cell extracts. (c) Predicted WT and MUT miR-136 binding sites in *MAT2A*. (d) Relative luciferase activities were measured in A549 and SPCA1 cells cotransfected with MAT2A-WT or MAT2A-MUT and miR-136 mimics or miR-NC by luciferase reporter gene assays. (e) Expression of *MAT2A* in lung cancer cells transfected with miR-136 mimics or miR-136 mimics combined with circ_0044516 overexpression plasmid. Data are presented as means ± SD. ^∗^*P* < 0.05; ^∗∗^*P* < 0.01; ^∗∗∗^*P* < 0.001.

We then searched for potential targets of miR-136 using http://starbase.sysu.edu.cn/. We identified *MAT2A* as a possible target of miR-136. *MAT2A* mRNA levels were enhanced in lung cancer (Figure 6(a)). We found that *MAT2A* was enriched by biotin-miR-136 (Figure 6(b)). WT-MAT2A and MUT-MAT2A were constructed (Figure 6(c)). Transfection with miR-136 mimics inhibited the activity of WT-MAT2A (Figure 6(d)). Additionally, circ_0044516 promoted *MAT2A* expression by sponging miR-136, as shown in Figure 6(e). Collectively, these assays demonstrated that *MAT2A* served as a direct target of miR-136.

### 3.7. Circ_0044516 Regulates Lung Cancer Cell Proliferation, Invasion, and Cancer Stem Cell Characteristics through Modulating miR-136 and *MAT2A*

**Figure 7:**
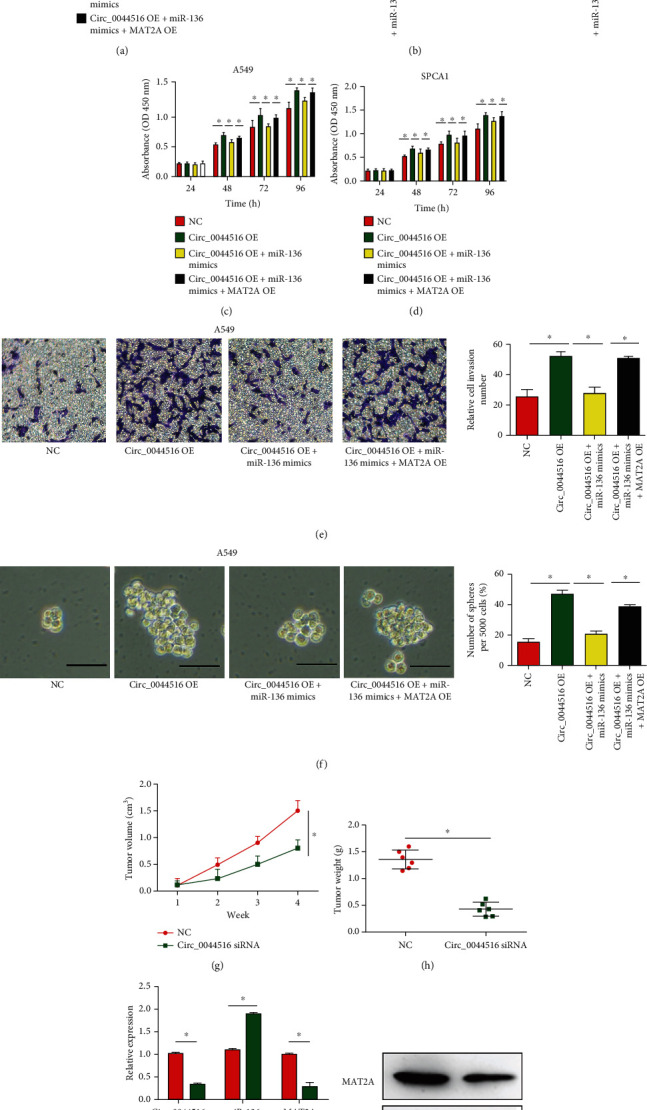
Circ_0044516 induced lung cancer cell proliferation, invasion, and cancer stem cell characteristics by modulating the miR-136/*MAT2A* pathway. (a, b) Expression of *MAT2A* in A549 and SPCA1 cells transfected with corresponding plasmids. (c, d) Cell proliferation of A549 and SPCA1 cells. (e) Cell invasion by A549 cells. (f) Tumor spheroid formation. Twelve 5-week old female BALB/c nude mice were injected with A549 cells infected with circ_0044516 siRNA (six mice) or NC (six mice). (g) Tumor volume. (h) Tumor weight. (i) Expression of circ_0044516, miR-136, and *MAT2A*. (j) Protein expression of MAT2A. Data are presented as means ± SD. ^∗^*P* < 0.05.

To determine whether circ_0044516 functions by modulating miR-136 and *MAT2A*, rescue assays were performed by transfection with miR-136 mimics or *MAT2A* OE plasmid inA549 and SPCA1 cells. Transfection efficiency was confirmed by analyzing *MAT2A* levels (Figures 7(a) and 7(b)). As shown, circ_0044516 overexpression increased proliferation, invasion, and cancer stem cell properties of A549 and SPCA1 cells (Figures 7(c)–7(f)). However, miR-136 mimics suppressed these processes, while *MAT2A* overexpression promoted the same. Moreover, circ_0044516 functions by modulating miR-136 and *MAT2A*. Subsequently, to evaluate whether circ_0044516 affected tumor growth *in vivo*, a human lung cancer xenograft model was established. We injected circ_0044516 siRNA or control-transfected A549 cells into nude mice. The results indicated that circ_0044516 siRNA suppressed lung cancer tumor volume in a time-dependent manner (Figure 7(g)). As shown, circ_0044516 downregulation reduced tumor weight (Figure 7(h)). Circ_0044516 and *MAT2A* levels were reduced in circ_0044516 siRNA-transfected tumor tissues, whereas miR-136 was increased (Figures 7(i) and 7(j)). In summary, these results demonstrated that circ_0044516 could function as a sponge for miR-136 to promote lung cancer progression by inducing *MAT2A* expression.

## 4. Discussion

circRNAs are important master regulators involved in multiple processes [16]. In recent years, the role of circRNAs in cancer progression has attracted considerable attention. Because of their cell- and tissue-specific unique molecular structures, circRNAs might have various regulatory functions in many biological processes and may represent superior diagnostic markers and therapeutic targets for cancer than linear transcripts [17]. Aberrant expression levels of circRNAs often lead to malignant behavior [18, 19]. However, the expression and role of most circRNAs in lung cancer development are unclear. Therefore, it is important to focus on the relationship between circRNAs and cancer. In the current study, a new role of circ_0044516 in lung cancer was demonstrated, and we reported its tumor-associated roles.

Dysregulated circRNAs are closely correlated with tumorigenesis in many cancers [20–22]. Previously, circ_0044516 was shown to promote prostate cancer development and progression [23]. Circ_0044516 promotes gastric cancer progression by modulating miR-149-5p and *HuR* [24]. Here, we demonstrated that circ_0044516 expression is upregulated in lung cancer. Thus, circ_0044516 could be a significant biomarker for lung cancer.

circRNAs can participate in various processes in multiple diseases [25–27]. For example, dysregulated circ_100876 induces esophageal squamous cell carcinoma progression [28]. In bladder cancer, circCDYL suppresses *c-MYC* to reduce cell proliferation and migration [29]. Circ-ITCH inhibits lung cancer proliferation by repressing Wnt/*β*-catenin [30]. Currently, we have shown that loss of circ_0044516 represses cell proliferation, blocks cell-cycle progression, and suppresses cell migration and invasion. In addition, cancer stem cells are considered to be responsible for tumor relapse. They may play significant roles in lung tumorigenesis [31]. In our study, we also demonstrated that silencing circ_0044516 obviously restrained the stemness of A549 and SPCA1 cells. Overexpression of circ_0044516 induces cancer stem cell properties.

Based on the ceRNA hypothesis, circRNA acts as a ceRNA to modulate miRNA target gene expression. To focus on the role of circ_0044516 in lung cancer, potential target miRNAs were predicted and miR-136 was identified. Circ_0014130 can reduce lung cancer cell apoptosis by sponging miR-136-5p and enhancing *BCL2* [32]. miR-136-5p is downregulated in patients with LUSC [33]. Additionally, NORAD promotes lung cancer cell proliferation by sponging miR-136-5p [34]. The association between circ_0044516 and miR-136 was demonstrated through luciferase reporter gene assays and RNA pull-down experiments. miR-136 expression is greatly reduced in lung cancer and acts as a tumor suppressor. Other miRNAs related to circ_0044516 require further investigation.

*MAT2A* was predicted and confirmed as a target of miR-136 in lung cancer. Bioinformatic analysis using TargetScan revealed that the biosynthesis of S-adenosylmethionine is a unique metabolic property of CSCs. Inhibition of *MAT2A* contributes to the repression of drug-resistant CSCs [35]. Overexpression of *MAT2A* has been shown in gastric cancer, and silencing of the *MAT2A* gene induces apoptosis and blocks cell cycle progression [36]. Nevertheless, the effects of *MAT2A* on lung cancer progression remains poorly understood. We observed that *MAT2A* was negatively modulated by miR-136 and positively regulated by circ_0044516. *MAT2A* reversed the effects of miR-136 on lung cancer cell growth, invasion, and cancer stem cell characteristics. In future studies, we would like to overexpress *MAT2A* alone to investigate whether it can induce a lung tumor model. These results further confirmed our hypothesis that circ_0044516 serves as a ceRNA for miR-136 to enhance *MAT2A* expression during lung cancer progression. However, the detailed mechanisms involved in the regulatory process of lung cancer require further elucidation.

Taken together, we identified a novel circRNA, circ_0044516, which plays an oncogenic role in lung cancer. We reported that circ_0044516 might sponge miR-136 to regulate *MAT2A* expression, leading to lung cancer progression. These data indicate a potential mechanism of action in lung cancer. However, the detailed mechanisms involved require further study.

## Figures and Tables

**Table 1:** Primers for real-time PCR.

Genes	Forward (5′-3′)	Reverse (5′-3′)
GAPDH	ACGGATTTGGTCGTATTGGG	TGATTTTGGAGG GATCTCGC
miR-136	ACUCCAUUUGUUUUGAUGAUGGA	UCCAUCAUCAAAACAAAUGGAGU
Circ_0044516	CGAGAGCATGACCGATGGAT	GCACCTTTAGGTCCAGGGAAT
U6	GCTTCGGCAGCACATATACTAAAAT	CGCTTCACGAATTTGCGTGTCAT
MAT2A	TAGCCTTGGAGCAACAGTCA	CCATTACGGCGTAGTTCTGC
CD133	CAATCTCCCTGTTGGTGATTTG	ATCACCAGGTAAGAACCCGGA
Sox2	GACAGTTACGCGCACATGAA	TAGGTCTG CGAGCTGGTCAT
Nanog	GTGATTTGTGGGCCTGA AGA	ACACAGCTGGGTGGAAGAGA
Oct4	GGTATTCAGCCAAACGA CCA	CACACTCGGACCACATCCTT

## Data Availability

The data used to support the findings of this study are included within the article.
